# Temporal Sculpting of Laser Pulses for Functional Engineering of Al_2_O_3_/AgO Films: From Structural Control to Enhanced Gas Sensing Performance

**DOI:** 10.3390/s25185836

**Published:** 2025-09-18

**Authors:** Doaa Yaseen Doohee, Abbas Azarian, Mohammad Reza Mozaffari

**Affiliations:** Department of Physics, University of Qom, Qom 3716146611, Iran; duaa_yaseen90@yahoo.com (D.Y.D.); abas_azarian@yahoo.com (A.A.)

**Keywords:** pulsed laser deposition (PLD), pulse duration, Al_2_O_3_/AgO films

## Abstract

This study examines the effects of laser pulse duration on the structural, morphological, optical, and gas-sensing characteristics of ${\mathrm{Al}}_{2}O_{3}$/AgO thin films deposited on glass substrates using pulsed laser deposition (PLD). Pulse durations of 10, 8, and 6 nanoseconds were achieved through optical lens modifications to control both energy density and laser spot size. X-ray diffraction (XRD) and atomic force microscopy (AFM) analyses showed a distinct reduction in both crystallite and grain sizes with decreasing pulse width, along with significant improvements in surface morphology refinement and film compactness. Hall effect measurements revealed a transition from n-type to p-type conductivity with decreasing pulse width, demonstrating increased hole concentration and reduced carrier mobility attributed to grain boundary scattering. Furthermore, current-voltage (I-V) characteristics demonstrated improved photoconductivity under illumination, with the most pronounced enhancement observed in samples prepared using longer pulse durations. Gas sensing measurements for ${\mathrm{NO}}_{2}$ and $H_{2}$S revealed enhanced sensitivity, improved response/recovery characteristics at 250 °C, with optimal performance achieved in films deposited using shorter pulse durations. This improvement is attributed to their larger surface area and higher density of active adsorption sites. Our results demonstrate a clear relationship between laser pulse parameters and the functional properties of ${\mathrm{Al}}_{2}O_{3}$/AgO films, providing valuable insights for optimizing deposition processes to develop advanced gas sensors.

## 1. Introduction

Semiconducting metal-oxide (SMO) chemiresistors are among the most widely studied platforms for gas sensing. Their signal arises from surface redox reactions that modulate the near-surface carrier concentration and, in turn, the film resistance. The basic idea—gas-induced changes in the electrical conductance of SMO materials—was articulated in the early 1950s [1].

Since then, SMO devices have been deployed alongside other solid-state technologies, notably electrochemical and catalytic-combustion sensors, as well as chemiresistive SMO sensors [2,3]. Performance gains have traditionally come from materials engineering strategies involving dopants, catalytic additives, binders/porogens, and electrode optimization [4] and, more recently, from nanoscale design strategies (e.g., heterojunctions, defect/oxygen-vacancy control, and oxide–carbon hybrids) aimed at improving sensitivity, response/recovery kinetics, and humidity tolerance [5,6,7,8].

Thin-film processing plays a comparably important role because it sets the microstructure (phase, crystallite/grain size, boundary density) and defect chemistry, both of which govern adsorption–reaction kinetics and charge transport. SMO layers have been prepared by pyrolysis, oxidation of metallic precursors, reactive sputtering, CVD, laser ablation, and e-beam evaporation, among other methods [9]. Within this landscape, pulsed laser deposition (PLD) offers rapid iteration and near-stoichiometric transfer from target to substrate. In this process, a pulsed beam ablates a solid target in a vacuum or in a controlled reactive ambient, generating a plasma plume that condenses into a film; early demonstrations with ruby lasers for semiconductor/dielectric coatings date to 1965 [10]. Recent work has revisited PLD for gas-sensing oxides, highlighting its ability to tune grain size, texture, and oxygen incorporation and to deliver uniform coatings at relevant length scales by adjusting laser and ambient parameters [11,12].

This study focuses on two high-priority analytes. Nitrogen dioxide (${\mathrm{NO}}_{2}$), largely emitted by traffic and industrial sources, contributes to photochemical smog and poses health risks even at relatively low concentrations [13]. Hydrogen sulfide ($H_{2}$S) is colorless, flammable, and highly toxic; chronic low-level exposure is associated with neurological and mucosal irritation, while higher doses are acutely hazardous [14]. Recent public-health assessments and air-quality reviews (2024–2025) underscore the need for sensitive, fast, and stable detection of both gases in realistic environments [15,16]. Against this backdrop, the present work examines PLD-grown SMO films as a process-centric route to co-tune microstructure and surface chemistry, situating the results within current trends in ${\mathrm{NO}}_{x}{\mathrm{/H}}_{2}$S sensing, as summarized in recent surveys [7,8]. Silver oxide (AgO) was selected as the active phase because it is a p-type metal-oxide semiconductor with well-documented reactivity toward ${\mathrm{NO}}_{2}$ and a strong affinity for $H_{2}$S. In the latter case, surface sulfidation (Ag → ${\mathrm{Ag}}_{2}$S) produces a large, rapid change in resistance that many $H_{2}$S sensors intentionally exploit. Embedding AgO in an ${\mathrm{Al}}_{2}O_{3}$ matrix pairs this redox responsiveness with the advantages of ${\mathrm{Al}}_{2}O_{3}$: chemical inertness, thermal stability, and its ability to disperse and cap the active grains, as well as to form controllable grain-boundary barriers that help stabilize the baseline and reduce humidity-induced drift. The composite, therefore, combines the catalytic/redox function of AgO with the mechanical/chemical robustness and high surface-to-volume ratio of ${\mathrm{Al}}_{2}O_{3}$ [17].

In this study, the ${\mathrm{Al}}_{2}O_{3}$:AgO ratio was kept fixed to cleanly isolate the effect of laser pulse duration on microstructure and charge transport—namely, grain size, boundary-barrier formation, and oxygen uptake. Composition tuning remains a valuable but independent lever that we plan to investigate separately. Many binary metal oxide systems display clear sensitivity maxima at specific mixing ratios due to percolation and heterojunction effects; well-known examples include optimized ${\mathrm{SnO}}_{2}$/ZnO and ${\mathrm{ZnO/SnO}}_{2}$ composites [18].

## 2. Experimental Procedure

### 2.1. Preparation of the ${\mathrm{Al}}_{2}O_{3}$/AgO Solid Target

A mixture was prepared by combining 0.2 g of silver oxide (AgO, 99.5% purity) with 0.8 g of aluminum oxide (${\mathrm{Al}}_{2}O_{3}$, 99% purity). The powder mixture was thoroughly homogenized and subsequently heated in a controlled-temperature oven at 120 °C for two hours to enhance material interaction. After the thermal treatment, the mixture was allowed to cool naturally to room temperature. It was then subjected to uniaxial pressing under a pressure of 5 tons to form a compact solid target with a diameter of 1.5 cm and a thickness of 0.5 cm, suitable for use in pulsed laser deposition (PLD) processes.

### 2.2. Preparation of the ${\mathrm{Al}}_{2}O_{3}$/AgO Thin Films

${\mathrm{Al}}_{2}O_{3}$/AgO thin films were deposited on glass substrates via pulsed laser deposition (PLD) at room temperature. A *Q*-switched Nd:YAG laser served as the excitation source, and the deposition chamber was operated at a pressure of 0.01 mbar. The nominal fluence at the target was maintained at 31 J/cm^2^. Three pulse-duration settings were investigated (τ=10, 8, and 6 ns). The baseline configuration employed τ=10 ns with a circular spot of $2W_{0}=2.5$ mm (where $W_{0}$ is the input beam waist radius).

**Figure 1:**
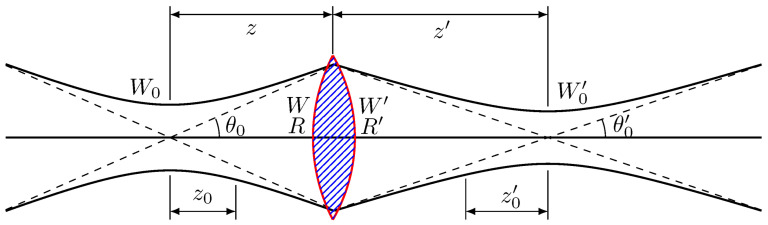
Transmission of a laser beam through a thin lens. τ is the pulse duration. $W_{0}$ is the beam waist radius before the lens, representing the narrowest point of the laser beam before it reaches the lens. $2W_{0}$ is called the spot size. $\theta_{0}$ is the divergence angle before the lens, indicating how much the beam spreads as it propagates away from the waist. $z_{0}$ is the Rayleigh range before the lens, defined as the distance from the beam waist to the point where the beam width approximately doubles. *R* is the radius of curvature of the wavefront before the lens, describing the curvature of the beam’s phase front. *z* represents the propagation distance from the waist to the lens. $W_{0}^{\prime }$ is the new beam waist after the lens, corresponding to the location of the new narrowest point of the beam after refocusing. $\theta_{0}^{\prime }$ is the divergence angle after the lens, showing how the beam spreads after passing through it. $z_{0}^{\prime }$ is the new Rayleigh range after the lens, analogous in concept to $z_{0}$, but for the transformed beam. $R^{\prime }$ is the radius of curvature of the wavefront after the lens, representing the curvature of the phase front post-lens. $z^{\prime }$ is the propagation distance from the lens to the new waist. *W* is the beam radius at the lens position. $W^{\prime }$ is the beam radius immediately after the lens.

As shown in Figure 1, when a laser beam passes through a thin lens placed at or near its focal length, the lens transforms the beam into a nearly collimated or weakly converging output, depending on the exact object distance relative to the focal length. In laser-beam optics, this transformation creates a new beam waist. Practically, if the beam is focused onto a screen, the plane where the smallest spot appears identifies the new waist location. The radius of the waist, $W_{0}^{\prime }$, can then be determined by measuring the radius of the smallest spot at that plane. Thus, a thin lens simultaneously shifts the waist and modifies its radius ($W_{0}^{\prime }$). These quantities may be obtained either from the standard Gaussian-beam Equations (1) and (2) or from direct measurement of the minimum spot size [19],

(1) $$W_{0}^{\prime }=W_{0}{\left[1+{\left(z_{0}/f\right)}^{2}\right]}^{1/2},$$

and

(2) $$\tau =\tau_{0}{\left[1+{\left(z_{0}/f\right)}^{2}\right]}^{1/2}.$$

Shorter effective pulse durations were realized by inserting an f=5 cm convex lens along the beam path and translating it relative to the laser output aperture. At lens positions of 4 cm and 5 cm, the spot diameters at the target were reduced to 2.0 mm and 1.5 mm, respectively. The pulse width was then calculated using $\tau /\tau_{0}=W_{0}^{\prime }/W_{0}={\left[1+{\left(z_{0}/f\right)}^{2}\right]}^{1/2}$, and the corresponding τ=8 ns and τ=6 ns settings were verified.

The laser was focused onto the ${\mathrm{Al}}_{2}O_{3}$/AgO target at a 45° incidence angle to promote efficient ablation. The substrates were mounted directly opposite the target, with their surfaces parallel to the target plane, to ensure uniform thickness across the deposited films.

### 2.3. Gas Sensor

The gas-sensing performance of the prepared ${\mathrm{Al}}_{2}O_{3}$/AgO thin-film samples was evaluated using a custom-built experimental setup. This system included sensors mounted with electrodes for signal detection, along with integrated pressure and temperature gauges to monitor environmental conditions during testing. For each measurement cycle, the start and end times of gas injection were precisely recorded to ensure consistency across experiments. The sensing tests were conducted at three different operating temperatures-40 °C, 150 °C, and 250 °C-to assess the thermal influence on sensor behavior. The sensors were exposed to controlled concentrations of two target gases: 150 ppm of nitrogen dioxide (${\mathrm{NO}}_{2}$) and 200 ppm of hydrogen sulfide ($H_{2}$S), to investigate the influence of laser energy density on gas response characteristics.

## 3. Results and Discussion

In pulsed laser deposition (PLD) of thin films, the laser pulse width (pulse duration, τ), defined as the temporal duration of the laser pulse, significantly influences the ablation efficiency of target material atoms. For longer pulse durations (tens of nanoseconds or more), the extended heating period allows for a more gradual energy transfer to the target material. This localized heating induces surface melting instead of direct atomic ablation. With longer pulse durations, the energy distribution occurs over an extended period, which reduces the ablation threshold energy while simultaneously raising the melting threshold. In contrast, shorter pulses (several nanoseconds or less) lead to a reduced heating time, resulting in a higher peak energy, which is the maximum energy reached during the pulse

(3) $$E_{\mathrm{rate}}=E/\tau$$

where $E_{\mathrm{rate}}$ denotes the energy deposition rate (J/s) in every pulse, *E* represents the pulse energy (J), and τ is the pulse width (s). This enables more efficient ablation of atoms from the surface without significant melting [20,21].

It is worth noting that the ablation threshold, defined as the minimum energy required to initiate atom ablation, decreases with shorter pulse widths, reducing the amount of material ablated. When this threshold is exceeded, the laser rapidly sublimates the material. Thus, shorter pulse widths prove more effective for atom ablation, particularly at higher energies. Under the same pulse energy conditions, shorter pulses tend to favor atom ablation, while longer pulses lead to surface melting due to the extended laser-material interaction time [20,22].

### 3.1. X-Ray Diffraction (XRD)

**Figure 2:**
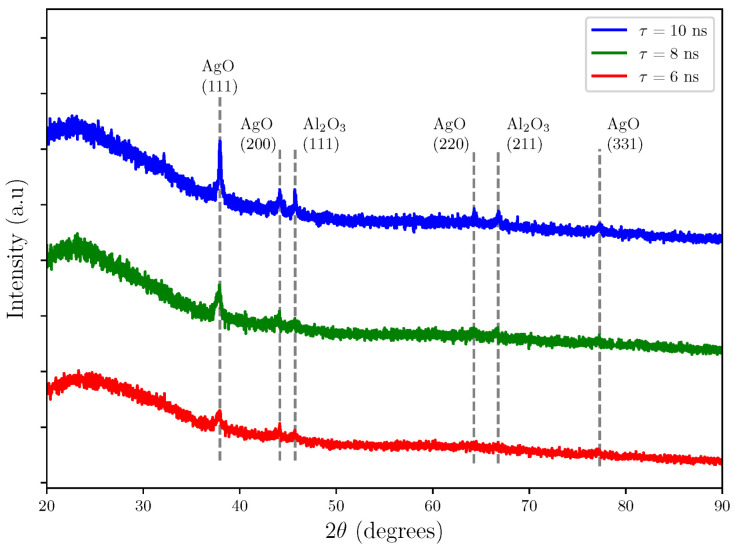
X-ray diffraction patterns of ${\mathrm{Al}}_{2}O_{3}$/AgO samples with a pulse duration of 10, 8, and 6 ns.

Figure 2 presents the X-ray diffraction patterns of ${\mathrm{Al}}_{2}O_{3}$/AgO thin films prepared by pulsed laser deposition on glass substrates at different laser pulse durations (6, 8, and 10 ns). The films have polycrystalline structures. For all pulse durations, the XRD patterns display multiple diffraction peaks corresponding to polycrystalline AgO ((111), (200), and (220) planes; JCPDS No. 76-1489) and polycrystalline ${\mathrm{Al}}_{2}O_{3}$ ((111) and (211) planes; JCPDS No. 01-1303) [23].

When using short laser pulses (10, 8, and 6) ns, the area of the laser spot (the area where the laser hits the target) is reduced to (0.0491, 0.0314, and 0.0177) ${\mathrm{cm}}^{2}$, respectively, if the pulse width is short. The laser’s energy is delivered to a small surface area in a very short time, causing extremely rapid heating. This rapid heating increases the pulse’s peak energy, especially with shorter pulse widths, since the same energy is concentrated into a briefer time. The concentrated energy overcomes the interatomic bonding forces in the material’s crystal lattice, resulting in fast and effective atom ejection from the surface. Consequently, the ablated atoms become smaller as the concentrated energy finely fragments the material, thereby reducing the crystal size of the deposited ${\mathrm{Al}}_{2}O_{3}$/AgO films. Therefore, as the pulse width decreases, the crystal size of the resulting ${\mathrm{Al}}_{2}O_{3}$/AgO films also decreases, as shown in Table 1. Figure 2, corresponding to this experiment, clearly illustrates the relationship between pulse width and crystal size [24].

### 3.2. Atomic Force Microscope (AFM)

**Figure 3:**
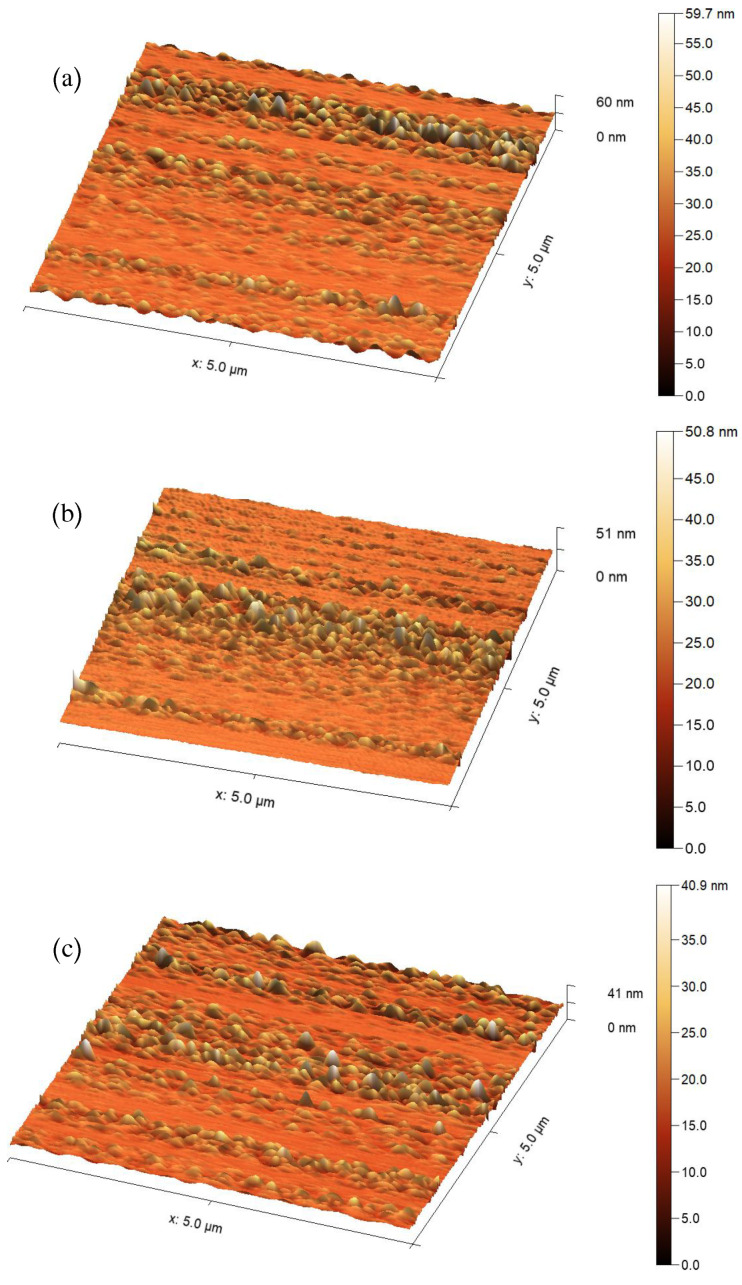
Atomic force microscopy 3D surface topography of ${\mathrm{Al}}_{2}O_{3}$/AgO samples at pulse durations of (**a**) 10 ns, (**b**) 8 ns, and (**c**) 6 ns.

Figure 3 displays 2D and 3D surface topologies of ${\mathrm{Al}}_{2}O_{3}$/AgO films on glass substrates, prepared via pulsed laser deposition at pulse durations of 10, 8, and 6 ns. The pulse width (10, 8, and 6 ns) significantly influences both the grain size and surface roughness of the resulting ${\mathrm{Al}}_{2}O_{3}$/AgO films. Longer pulse widths (10 ns) distribute energy over a longer duration, reducing energy concentration and causing slower heating of the ${\mathrm{Al}}_{2}O_{3}$/AgO target. The resulting gradual cooling promotes grain growth (27.19 nm, Table 2), yielding larger grains and a rougher surface morphology. Conversely, shorter pulse widths (8 and 6 ns) increase the peak pulse energy by concentrating the same total energy into briefer durations. This produces more intense, rapid heating of the ${\mathrm{Al}}_{2}O_{3}$/AgO target, enhancing atomic mobility and deposition efficiency. The higher peak energy consequently reduces grain size (23.95 and 17.87 nm), causing finer material fragmentation during deposition and ultimately yielding a smoother surface.

### 3.3. Field Emission Scanning Electron Microscopes (FE-SEM)

**Figure 4:**
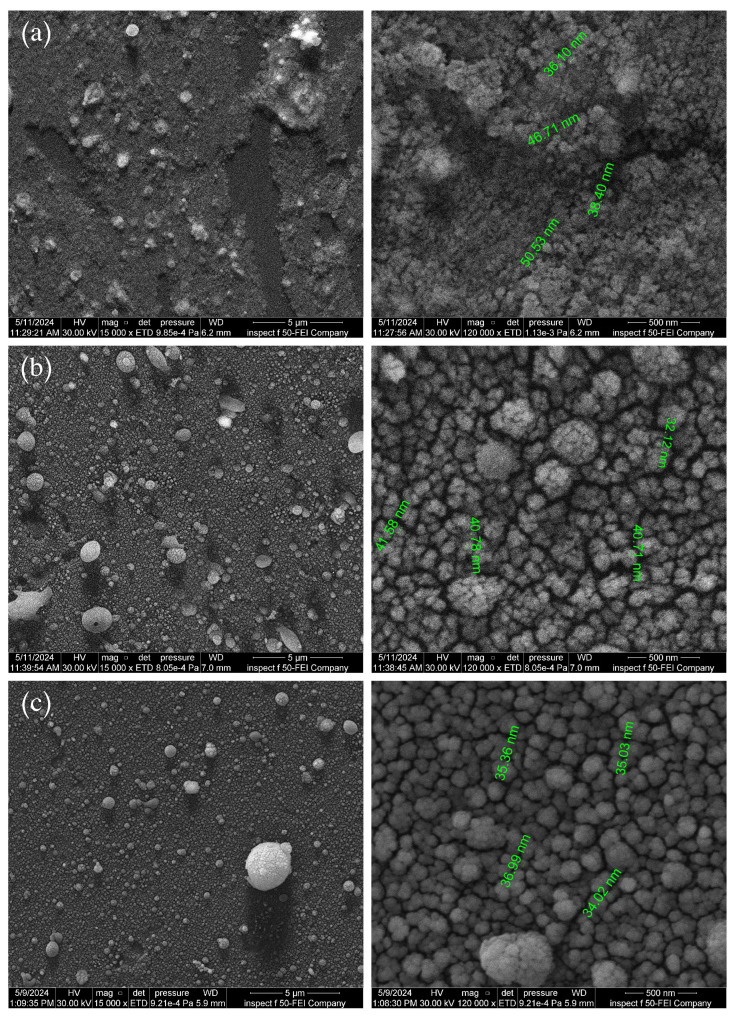
FE-SEM surface morphology of ${\mathrm{Al}}_{2}O_{3}$/AgO samples at pulse durations of (**a**) 10 ns, (**b**) 8 ns, and (**c**) 6 ns.

FE-SEM top-view images and corresponding particle size distributions of ${\mathrm{Al}}_{2}O_{3}$/AgO films on glass substrates at laser pulse durations of 10, 8, and 6 ns are presented in Figure 4. As the laser pulse duration decreases, the energy is delivered over a shorter period, increasing the peak energy and causing more intense and rapid heating of the target. This leads to violent evaporation and the formation of a denser plasma, which favors the production of smaller particles due to rapid material dispersion [25]. The short pulse creates a sharp temperature gradient that instantly evaporates material, generating small vapor droplets that rapidly condense on the cooler substrate surface. Consequently, smaller particles form as the aggregation time is minimized.

As pulse duration decreases from 10 ns to 8 ns and then to 6 ns, both plasma density and species kinetic energy increase, enhancing the production of finer particles [25]. Moreover, rapid plasma cooling inhibits particle growth and coalescence, preserving their small size. Consequently, film molecular sizes decrease to 42.94 nm, 35.35 nm, and 30.10 nm for pulse durations of 10, 8, and 6 ns, respectively.

### 3.4. Cross-Section FE-SEM Images

**Figure 5:**
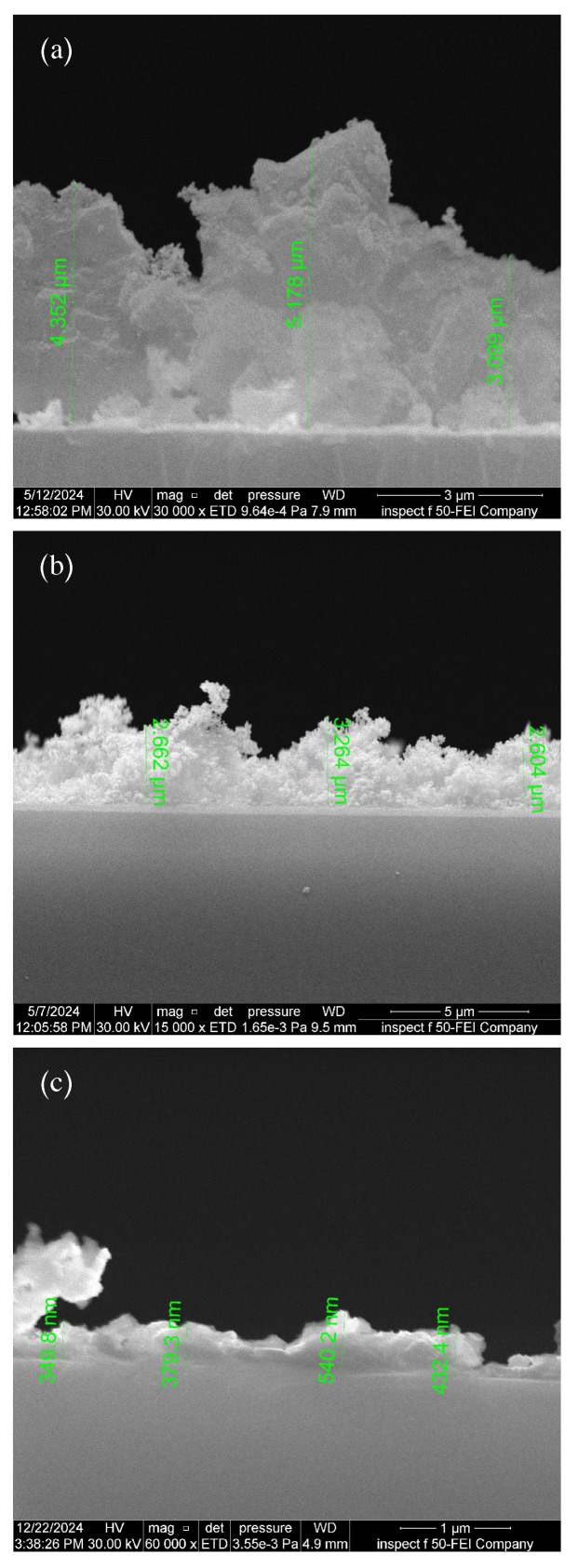
FE-SEM cross-sections of ${\mathrm{Al}}_{2}O_{3}$/AgO films deposited at pulse durations of (**a**) 10 ns, (**b**) 8 ns, and (**c**) 6 ns.

The thickness of thin films deposited on glass substrates at room temperature using PLD with different pulse durations (10, 8, and 6 ns) was determined through tomographic analysis via field-emission scanning electron microscopy (FE-SEM), as shown in Figure 5. When the laser pulse width decreases from 10 ns to 8 ns and then to 6 ns, the laser spot area (the irradiation zone on the target) correspondingly reduces to $0.0491{\mathrm{cm}}^{2}$, $0.0314{\mathrm{cm}}^{2}$, and $0.0177{\mathrm{cm}}^{2}$, respectively. This reduction critically determines both the resulting plasma properties and the deposition pattern on the substrate.

When using a smaller laser spot area ($0.0177{\mathrm{cm}}^{2}$), the particle emission angle from the target increases significantly, producing both a wider plasma plume and more extensive visible expansion. This occurs because the small spot acts as a point energy source, concentrating energy in a limited area and causing particles to disperse at wider angles. Conversely, with a larger laser spot ($0.0491{\mathrm{cm}}^{2}$), the spread angle is smaller, a broader surface of the target is heated, leading to a higher number of emitted particles. This produces a denser, more narrowly confined plasma plume, where frequent particle collisions limit dispersion and focus the plume direction [24]. Consequently, as the laser pulse width decreases from 10 ns to 8 ns to 6 ns, the resulting film thickness reduces to 4.2097μm, 2.8434 μm, and 0.425μm, respectively.

The laser spot area significantly influences the film thickness distribution across the substrate. A smaller spot area produces wider particle dispersion, creating greater thickness variation in the deposited film, with decreased thickness at the edges and increased accumulation at the center. For monoelemental targets, this variation primarily influences film thickness uniformity. However, with multielemental targets like ${\mathrm{Al}}_{2}O_{3}$/AgO, the differential emission angles and broader particle dispersion can produce chemically heterogeneous films. In such cases, elements evaporate disproportionately and deposit non-uniformly across the substrate surface [24].

### 3.5. Energy Dispersive Spectroscopy (EDS)

**Figure 6:**
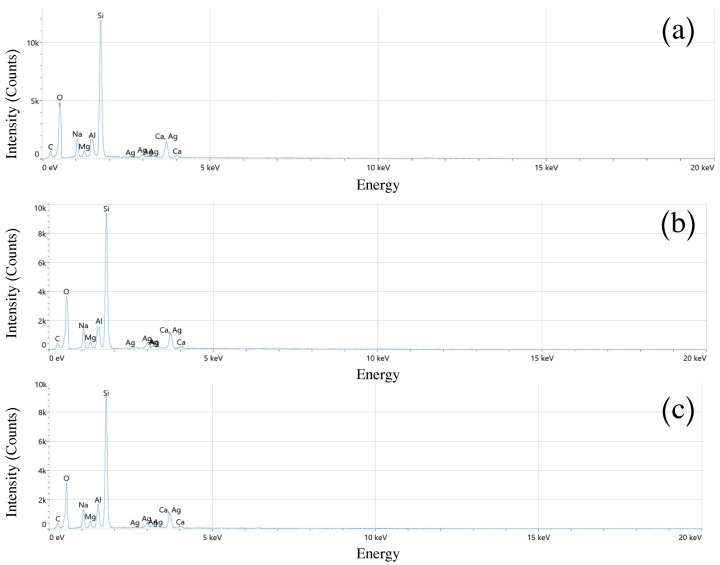
Energy-dispersive spectroscopy (EDS) analysis of ${\mathrm{Al}}_{2}O_{3}$/AgO samples at pulse durations of (**a**) 10 ns, (**b**) 8 ns, and (**c**) 6 ns.

The chemical composition of ${\mathrm{Al}}_{2}O_{3}$/AgO films prepared at different pulse durations (10, 8, and 6 ns) was analyzed using energy-dispersive spectroscopy (EDS), as shown in Figure 6. EDS analysis confirmed the presence of all expected elements in the samples, with average weight and atomic percentages as listed in Table 3. The results demonstrate that the prepared ${\mathrm{Al}}_{2}O_{2}$/AgO films exhibit high purity without detectable by-products. The peaks observed in Figure 6 (Si, O, C, Na, Mg, and Ca) originate from the glass substrate used for deposition. The manufacturing of glass substrates involves materials of silicon Dioxide (${\mathrm{SiO}}_{2}$): providing structural integrity and thermal stability, Sodium carbonate (${\mathrm{Na}}_{2}{\mathrm{CO}}_{3}$): Which is used to reduce the melting temperature of the glass, making the manufacturing process easier, and magnesium Oxide (MgO) and Calcium Oxide (CaO): These oxides, contribute to the glass’s durability and thermal expansion characteristics [26].

As the pulse width decreases from 10 ns to 8 ns and then to 6 ns, the evaporation process intensifies significantly because the ${\mathrm{Al}}_{2}O_{3}$/AgO target material undergoes extremely rapid heating within a confined timeframe. This generates a dense plasma composed of Al, Ag, and O in varying stoichiometric ratios. The resulting increase in plasma density at shorter pulse widths promotes frequent high-energy collisions between ions and particles within the plasma. These collisions significantly enhance the oxygen concentration in the plasma via copper reoxidation, ultimately producing an oxygen-rich film upon substrate deposition. Consequently, when using shorter pulses (6 ns), EDS analysis consistently shows higher oxygen content relative to aluminum and silver [27] as quantified in Table 3.

In contrast, a 10 ns pulse width produces relatively slower evaporation, resulting in lower plasma intensity and consequently less dense plasma formation. In this regime, the O−Al−Ag interactions are less intense, producing films with higher metal-to-oxygen ratios. Consequently, EDS analysis shows greater Al and Ag content at 10 ns pulse widths compared with 6 ns conditions, where elevated oxidation rates yield higher oxygen concentrations [27].

Furthermore, shorter pulse widths reduce the laser spot area on the target surface (as previously established), producing a wider plasma plume dispersion across the substrate. This effect yields thinner, more compositionally heterogeneous deposited layers. Practically, this results in thinner film deposition at 6 ns pulse widths, where the expanded vapor dispersion area and reduced particle density contrast with the more concentrated deposition observed at 10 ns.

### 3.6. Optical Properties

**Figure 7:**
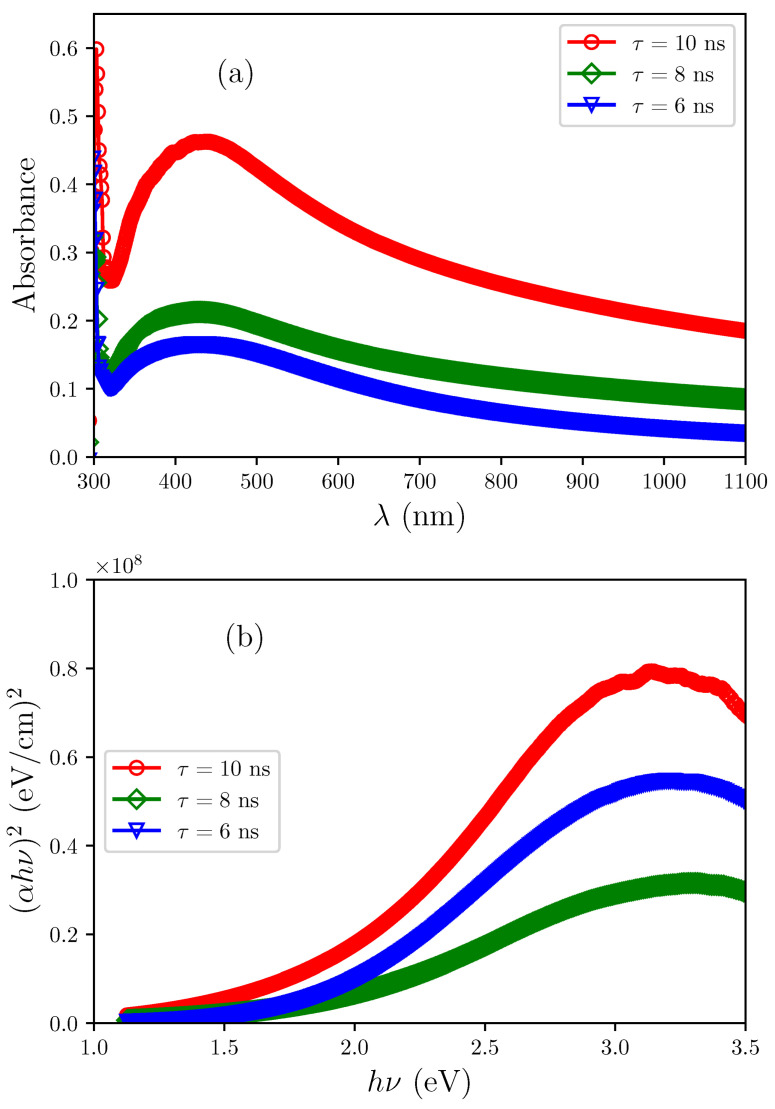
(**a**) Absorbance versus wavelength and (**b**) ${\left(\alpha h\nu \right)}^{2}$ versus photon energy (hν) for ${\mathrm{Al}}_{2}O_{3}$/AgO thin films at pulse durations of 10 ns, 8 ns, and 6 ns.

Figure 7a shows the absorption spectrum of ${\mathrm{Al}}_{2}O_{3}$/AgO films prepared by PLD deposited with different pulse durations (10, 8, and 6 ns). The spectra show strong absorbance in the UV region, which decreases sharply across the visible range. This gradual absorbance reduction with increasing wavelength suggests low crystallinity in all prepared samples. Reducing the pulse width from 10 ns to 8 ns and then to 6 ns significantly modifies the absorbance characteristics of the deposited thin films. This effect starts with a shift of the absorption edge toward shorter wavelengths, indicating an increase in the energy gap of the film. The optical band gap decreases from 1.821 to 1.855 and then to 1.902 eV, as shown in Figure 7b, when the width of the beam is reduced from 10, 8, to 6 ns. This phenomenon arises from quantum size effects, where the reduction in particle size due to the shorter pulse width intensifies the quantum constraints experienced by the electrons. Consequently, electron transitions between energy levels require higher-energy photons corresponding to shorter wavelengths.

Alongside this spectral shift, the overall absorbance progressively decreases with reducing pulse width. This reduction primarily results from decreased film thickness, which reduces the light-absorbing material volume.

### 3.7. Hall Effect

The Hall coefficient ($R_{H}$), carrier concentration ($n_{H}$), conductivity type, and Hall mobility ($\mu_{H}$) were measured for ${\mathrm{Al}}_{2}O_{3}$/AgO films prepared at different pulse durations (10, 8, and 6 ns) using PLD. Table 4 clearly shows a transition in conductivity type from n-type (10 ns pulse) to p-type (8 and 6 ns pulses). All samples prepared with shorter pulses exhibited positive Hall coefficients, which confirms p-type behavior. This transition correlates strongly with the compositional trends observed in the EDS data (Table 3), where oxygen concentration increases as silver and aluminum contents decrease with reducing pulse duration. The enhanced oxygen incorporation modifies the chemical bonding environment within the ${\mathrm{Al}}_{2}O_{3}$/AgO matrix, introducing additional oxygen-related acceptor states [28]. These states capture electrons from the valence band, thereby generating electron holes that act as majority carriers in p-type semiconductors. This mechanism explains both the observed conductivity transition and the concurrent widening of the optical bandgap (Figure 7b)—a well-known consequence of oxygen enrichment and quantum confinement effects in oxide semiconductors.

The simultaneous reduction in carrier mobility and carrier density, as listed in Table 5, can be attributed to structural refinements induced by shorter pulse durations. Specifically, XRD and AFM analyses (Table 1 and Table 2) demonstrate a reduction in both crystallite and grain size. This increase in grain boundary density introduces additional scattering centers, leading to enhanced grain boundary potential barriers [29], which limit the mean free path of charge carriers and reduce mobility. Although mobility degradation is typically detrimental to electronic transport, it indirectly benefits gas sensing performance in this case; the higher density of grain boundaries and surface defects provides more active adsorption sites for gas molecules, thereby enhancing sensor sensitivity [30]. Thus, the pulse-duration-induced transition from n- to p-type conductivity in ${\mathrm{Al}}_{2}O_{3}$/AgO films is not solely an electrical phenomenon but rather a coupled structural–chemical effect, driven by oxygen incorporation, lattice defect formation, and grain boundary engineering [31].

### 3.8. Current-Voltage Characteristics

**Figure 8:**
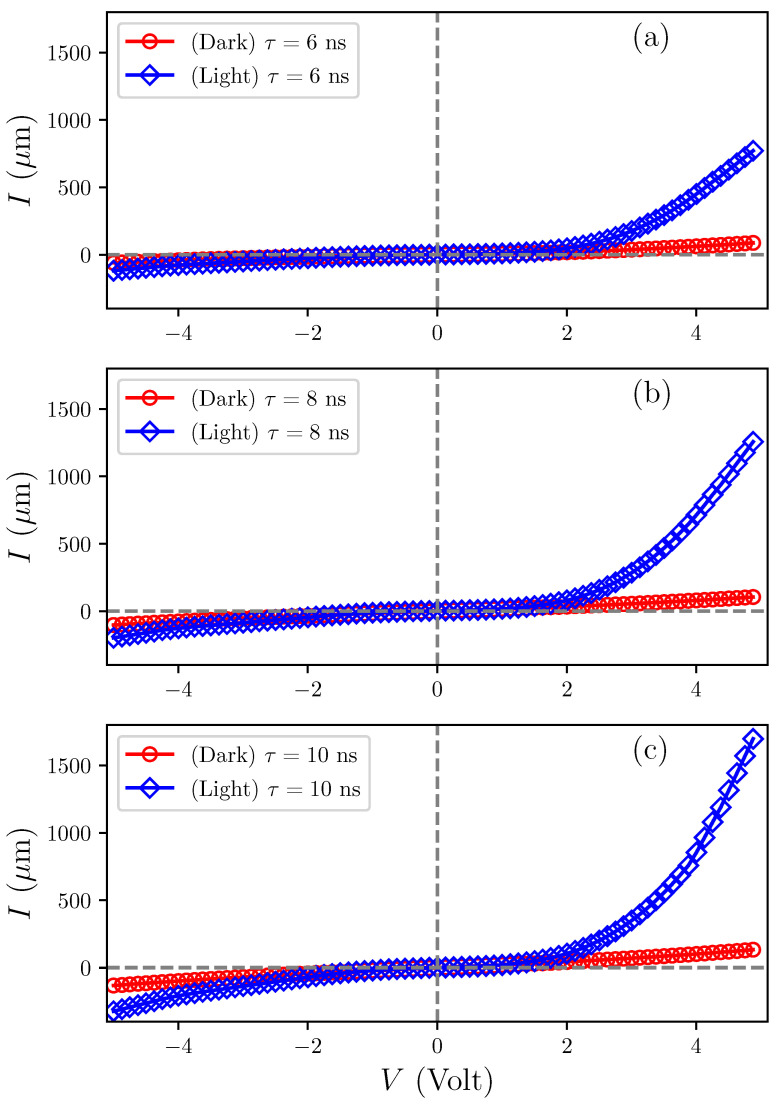
Current-voltage (I-V) characteristics of ${\mathrm{Al}}_{2}O_{3}$/AgO films with pulse durations of (**a**) 10 ns, (**b**) 8 ns, and (**c**) 6 ns.

Figure 8 shows the results obtained by changing the current-voltage of ${\mathrm{Al}}_{2}O_{3}$/AgO films prepared by PLD deposition with pulse durations of 10 ns, 8 ns, and 6 ns. All measurements were performed at a constant temperature of 40 K. The current-voltage (I-V) characteristics of these films were methodically analyzed under dark conditions (black curve) and under illumination (red curve) at a specified light intensity, as detailed in Figure 8. The observations revealed a significant enhancement in photoconductivity under illumination compared with dark conditions. This increase is mainly due to the creation of more free charge carriers (electrons and holes). The sample with a laser pulse duration of τ=10 ns showed the highest recorded forward bias current. Increasing the applied potential difference caused the current through the film to rise, with the forward bias current exhibiting near-exponential behavior. This indicates the dominance of the propagation current, originating from the random movement of charge carriers (electrons and holes) from regions of high concentration to regions of lower concentration according to Fick’s Law of Diffusion, over the recombination current, which results from electron-hole recombination, thereby reducing the number of free carriers and diminishing the overall current.

The reduction in the built-in voltage and the narrowing of the depletion region were attributed to the increased applied voltage, which enhanced the injection of majority carriers. In comparison, the samples with pulse durations of τ=8 ns and 6 ns recorded lower forward bias current values than the τ=10 ns sample. Additionally, as shown in Figure 8, the reverse bias current appeared across two regions. Initially, low current values were observed, resulting from the expansion of the depletion region and the consequent decrease in carrier concentration.

The observed decrease in both the ${\mathrm{Al}}_{2}O_{3}$/AgO film’s crystal size (by XRD) and grain size (by AFM) with reduced pulse width suggests increased crystal defects and larger intergranular gaps in the film. This increase in defects creates more electron-trapping sites that impede conduction, thereby reducing the electrical conductivity of the ${\mathrm{Al}}_{2}O_{3}$/AgO film. Additionally, as the crystal size decreases, the energy levels become more widely spaced, increasing the energy required for electron transitions from the valence band to the conduction band. As a result, fewer electrons can reach the conduction band, reducing the population of effective charge carriers and consequently limiting the electrical conductivity of the ${\mathrm{Al}}_{2}O_{3}$/AgO film.

### 3.9. Sensing Characteristics of ${\mathrm{Al}}_{2}O_{3}$/AgO Towards Concentration of ${\mathrm{NO}}_{2}$ and $H_{2}S$ Gases

**Figure 9:**
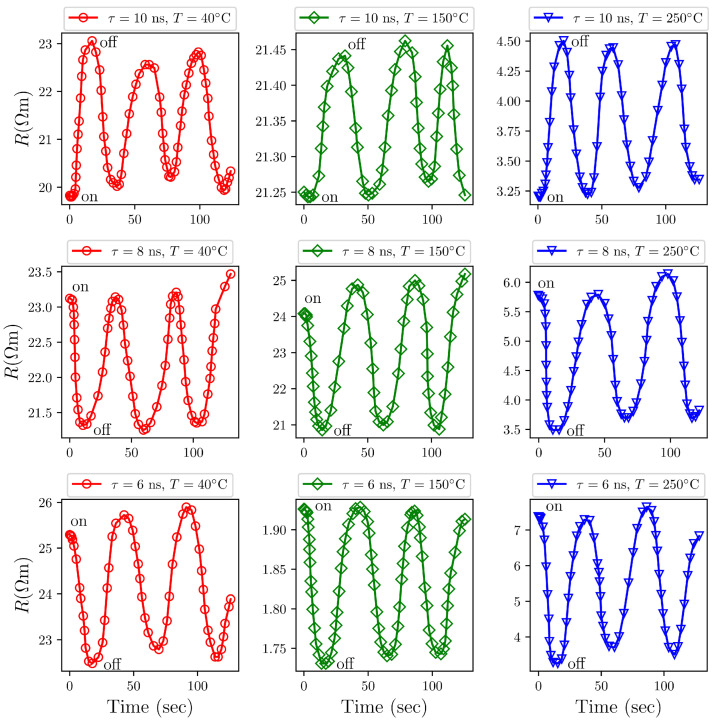
Resistance variation in ${\mathrm{Al}}_{2}O_{3}$/AgO thin films deposited under different laser pulse durations (10 ns, 8 ns, and 6 ns) when exposed to 150 ppm ${\mathrm{NO}}_{2}$ gas at various operating temperatures.

**Figure 10:**
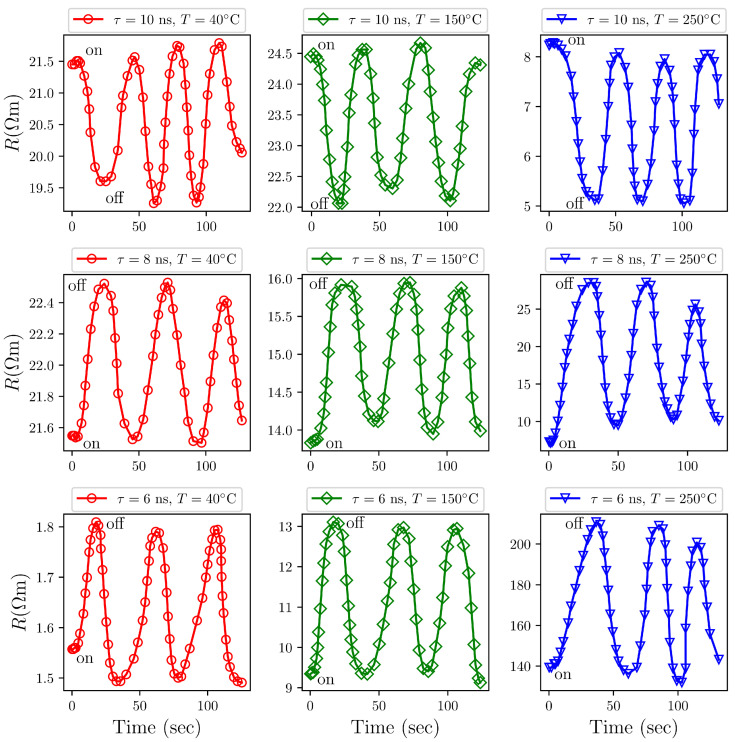
Resistance variation in ${\mathrm{Al}}_{2}O_{3}$/AgO thin films deposited under different laser pulse durations (6 ns, 8 ns, and 10 ns) when exposed to 200 ppm $H_{2}$S gas at various operating temperatures.

To evaluate the gas-sensing performance, we investigated the temporal resistance variations in ${\mathrm{Al}}_{2}O_{3}$/AgO thin films upon exposure to both $H_{2}$S (a reducing gas) and ${\mathrm{NO}}_{2}$ (an oxidizing gas). We deposited the films onto glass substrates via PLD using lasers with pulse durations of 10 ns, 8 ns, and 6 ns. The operating temperature range was 40–250 °C, and the experimental results are summarized in Figure 9 and Figure 10.

#### 3.9.1. Sensing Mechanism

A sample of ${\mathrm{Al}}_{2}O_{3}$/AgO that deposits with τ=10 ns showed n-type semiconductor characteristics, with resistance increasing under ${\mathrm{NO}}_{2}$ exposure and decreasing under $H_{2}$S exposure. This behavior is consistent with the typical sensing mechanism of metal oxide semiconductors, which mainly relies on changes in surface resistance. Atmospheric oxygen molecules adsorb onto the film surface, where they become ionized by capturing electrons from the conduction band, forming oxygen species ($O_{2}^{-}$ and $O^{-}$). This process creates a surface depletion layer and generates potential barriers that hinder charge carrier transport between nanoparticles.

When exposed to reducing gases such as $H_{2}$S, the chemisorbed oxygen species react with gas molecules, releasing previously trapped electrons back into the conduction band and consequently reducing resistance. In contrast, oxidizing gases like ${\mathrm{NO}}_{2}$ extract additional electrons from the material, which both thicken the depletion layer and increase the resistance. These dynamic alterations in surface charge density directly influence the electrical conductivity of the sensor devices [32].

The gas sensing mechanism in p-type semiconductor thin films is primarily governed by surface interactions that modulate the majority charge carrier (hole) concentration. When these films are exposed to ${\mathrm{NO}}_{2}$ gas (a strong oxidizing agent with high electron affinity), chemical reactions occur between the gas molecules and the semiconductor’s active surface. This interaction causes electron extraction from the surface, which increases hole concentration through valence band electron removal. As a result, the film’s electrical resistance decreases [33]. Conversely, when exposed to $H_{2}$S gas (a reducing electron donor), the film undergoes surface reactions that add electrons to the semiconductor. These electrons recombine with existing holes, thereby reducing hole concentration. This decrease in positive charge carriers leads to a significant increase in the film’s electrical resistance. These resistance changes stem directly from gas–semiconductor interactions, demonstrating the core mechanism through which p-type materials detect gas identity and concentration in sensing applications.

#### 3.9.2. Response and Recovery Times

Table 5 presents the quantitative determination of target gas sensitivity, response time, and recovery time for ${\mathrm{Al}}_{2}O_{3}$/AgO sensor architectures fabricated using different laser pulse durations (10 ns, 8 ns, 6 ns) under various thermal conditions. We consistently observed that recovery times were longer than response times for all pulse durations. This pattern indicates the slower desorption kinetics governing gas molecule release from the active surface post-exposure. Conversely, the response phase, which involves rapid gas adsorption, develops faster because of stronger driving forces present during initial exposure.

#### 3.9.3. Effect of Sensor Operation Temperature

At low temperatures (40 °C), the limited kinetic energy of gas molecules reduces their interaction rate with the ${\mathrm{Al}}_{2}O_{3}$/AgO surface, leading to slower response times and lower sensitivity, as shown in Table 5. Increasing temperature elevates molecular kinetic energy, which enhances both adsorption and surface reaction rates on the ${\mathrm{Al}}_{2}O_{3}$/AgO film, thereby improving sensor sensitivity.

For ${\mathrm{NO}}_{2}$ (an oxidizing gas), elevated temperatures promote electron extraction from the ${\mathrm{Al}}_{2}O_{3}$/AgO surface, increasing hole concentration and causing a more significant resistance decrease. ${\mathrm{NO}}_{2}$ undergoes direct surface adsorption. In this case, the energy barriers at grain boundary surfaces are severely changed due to additional charges. Ultimately, the process increases the hole accumulation layer on the ${\mathrm{Al}}_{2}O_{3}$/AgO surface, i.e., increasing the electrical conductivity, enhancing the sensor response.

When increasing the temperature to 150 °C, gas–surface interactions cause higher resistivity during ${\mathrm{NO}}_{2}$ exposure [34], as confirmed by the experimental results in Table 5. The table also indicates that 250 °C is the optimal operating temperature for ${\mathrm{Al}}_{2}O_{3}$/AgO sensors.

For $H_{2}$S (a reducing gas), elevated temperatures enhance electron donation to the semiconductor. These donated electrons recombine with holes, reducing hole concentration and consequently increasing resistance significantly. Thus, the ${\mathrm{Al}}_{2}O_{3}$/AgO sensor achieves maximum sensitivity at an optimal operating temperature of 250 °C.

Therefore, the gas sensitivity of p-type semiconductor films to ${\mathrm{NO}}_{2}$ and $H_{2}$S depends critically on maintaining an optimal temperature range. Moderate to moderately high temperatures typically enhance both surface interactions and sensor performance.

#### 3.9.4. Effect of Decreasing Pulse Duration on Film Sensitivity

The sensitivity definition varies depending on the gas type (oxidizing or reducing). For reducing gases, sensitivity is defined as

(4) $$S=\frac{R_{g}-R_{a}}{R_{g}}\times 100\%$$

where $R_{a}$ and $R_{g}$ represent the resistance in air and gas, respectively. For oxidizing gases, the sensitivity is defined as

(5) $$S=\frac{R_{a}-R_{g}}{R_{a}}\times 100\%$$

#### Exposure to ${\mathrm{NO}}_{2}$ Gas

The sensing mechanism of ${\mathrm{Al}}_{2}O_{3}$/AgO exhibits a conductivity transition from n-type to p-type behavior due to preparation conditions. This transition relates to gas species ionosorption on the surface, where charge transfer between gas and surface molecules alters the electrical conductance [35]. Figure 9 shows decreased resistance in p-type ${\mathrm{Al}}_{2}O_{3}$/AgO films during ${\mathrm{NO}}_{2}$ exposure, followed by resistance recovery when the gas flow is terminated. This behavior results from interactions between the target gas and the metal oxide film surface, primarily mediated by adsorbed oxygen ions. These interactions alter the material’s charge carrier concentration, consequently modifying its conductivity (or resistivity).

Figure 9 specifically shows resistance decreasing during gas exposure and rapidly increasing after gas removal, exhibiting classic p-type semiconductor response patterns. In p-type materials, conduction primarily occurs through positive holes as charge carriers. Thus, when an oxidizing gas like ${\mathrm{NO}}_{2}$ adsorbs onto the surface, especially at grain boundaries, it interacts with oxygen ions, reducing the hole concentration. As a result, the conductivity decreases, and the material’s resistance increases once gas exposure ceases [36].

Nitrogen dioxide (${\mathrm{NO}}_{2}$) is a strong oxidizing gas. When it interacts with a p-type film such as ${\mathrm{Al}}_{2}O_{3}$/AgO, it withdraws electrons from the film surface, increasing the hole concentration, which are the majority charge carriers in p-type materials. This electron-withdrawing behavior enhances the film’s conductivity, which in turn, increases its gas sensitivity.

As the laser pulse duration decreases from 10 to 6 nanoseconds, smaller crystallites and grains of ${\mathrm{Al}}_{2}O_{3}$/AgO are deposited, as shown by XRD and AFM data in Table 1 and Table 2. The resulting reduction in crystallite and particle size increases the surface-to-volume ratio, leading to more chemically active sites available for gas interaction. This consequently enhances the sensitivity values, as shown in Table 5. The slight decrease in surface roughness indicates improved film compactness, which reduces structural defects and enhances selectivity toward oxidizing gases such as ${\mathrm{NO}}_{2}$.

#### Exposure to H_2_S Gas

${\mathrm{Al}}_{2}O_{3}$/AgO, characterized as a p-type semiconductor, adsorbs oxygen molecules ($O_{2}$) from the surrounding air. Due to the high oxidizing ability of $O_{2}$, these oxygen molecules capture electrons from the valence band, forming surface oxygen ions ($O^{-}$) via the reaction, $O_{2}$(ads)$\;+\;{2e}^{-}$ → 2O^−^(ads) [37,38].

This electron transfer induces the generation of holes in the valence band of the ${\mathrm{Al}}_{2}O_{2}$/AgO material. The resulting surface oxygen ions ($O^{-}$) are highly reactive. The interaction of H_2_S gas with adsorbed oxygen species ($O^{-}$) on the ${\mathrm{Al}}_{2}O_{2}$/AgO surface results in the reaction, $H_{2}$S(ads) $+\;3O^{-}$(ads) → H_2_O + SO_2_ + 2e^−^ [39].

This reaction releases electrons, which subsequently recombine with holes in the valence band, increasing the electrical resistance of the ${\mathrm{Al}}_{2}O_{3}$/AgO sensing films as shown in Figure 10.

Additionally, another chemical interaction may occur [40]. Although ${\mathrm{Al}}_{2}O_{3}$/AgO remains largely inert toward $H_{2}$S, AgO can react as H_2_S(ads) + AgO → Ag_2_S + H_2_O. This reaction produces a thin ${\mathrm{Ag}}_{2}$S surface layer on AgO grains, which significantly lowers the AgO resistance. Consequently, the initial resistance increase observed during sensing is primarily due to the reaction, $O_{2}$(ads) +2e^−^ → 2O^−^(ads), while the subsequent ${\mathrm{Ag}}_{2}$S formation from reaction, $H_{2}$S(ads) $+\;3O^{-}$(ads) → $H_{2}$O + SO_2_ + 2e^−^, causes a significant decrease in the sensor’s resistance.

The smaller crystallite and particle sizes of ${\mathrm{Al}}_{2}O_{3}$/AgO produced by shorter laser pulses (6 and 8 ns), as evidenced by XRD and AFM data in Table 1 and Table 2, increase grain boundary density. These boundaries serve as preferential sites for gas adsorption and surface reactions. Furthermore, the smaller nanoparticles exhibit higher surface energy. This energy results from the unstable state of surface atoms, which lack complete molecular bonding compared with atoms in the material’s bulk. These surface atoms interact with their surroundings to achieve greater stability, enhancing their chemical reactivity with reducing gases such as $H_{2}$S. This effect improves the sensitivity of ${\mathrm{Al}}_{2}O_{3}$/AgO films, as demonstrated in Table 5.

## 4. Benchmark Comparison of PLD-Grown ${\mathrm{Al}}_{2}O_{3}$/AgO Films Versus Contemporary Thin-Film Platforms for ${\mathrm{NO}}_{2}$ and $H_{2}$S Gas Sensing

The PLD ${\mathrm{Al}}_{2}O_{3}$/AgO films, tuned by shortening the laser pulse to densify intergranular interfaces, exhibited consistently fast chemiresistive behavior at 250 °C. Within the high-dose window explored here, the film exhibited a response of 55.56% to 150 ppm ${\mathrm{NO}}_{2}$ with 11.61 s response and 20.43 s recovery times, and a response of 74.20% to 200 ppm $H_{2}$S with 24.84 s response and a best-in-set 16.02 s recovery time (total cycle time ≈40.86 s).

As shown in Table 6, for $H_{2}$S detection, the ${\mathrm{Al}}_{2}O_{2}$/AgO sensor offers the most advantageous time-domain trade-off among representative comparators. A ${\mathrm{CuO/SnO}}_{2}$ p–n heterojunction (tested at 50 ppm and 200 °C) reaches a higher nominal response (85.71%) but is much slower (response: 100 s/recovery: 109 s). NiO (a single-phase p-type metal oxide; tested at 200 ppm and 400 °C) shows only a 28.8% response and still lags in kinetics (response: 108 s/recovery: 47 s) despite the higher operating temperature. A hybrid ${\mathrm{Ag/WO}}_{2}$/rGO film (100 ppm, 150 °C) has the fastest rise time (8 s) but recovers more slowly (38 s), yielding a total cycle time (46 s) longer than that of the ${\mathrm{Al}}_{2}O_{3}$/AgO film (≈40.86 s). Overall, the PLD ${\mathrm{Al}}_{2}O_{3}$/AgO film surpasses the composite p-n, single-phase, and hybrid multi-phase references in $H_{2}$S time-domain performance, while avoiding the need for an extra activation pathway [41].

As shown in Table 6, for ${\mathrm{NO}}_{2}$ at concentrations ≥100 ppm, the ${\mathrm{Al}}_{2}O_{3}$/AgO sensor likewise prioritizes reversible, seconds-scale cycling. Microporous NiO (tested at 100 ppm and 200 °C) offers a 45.6% response with a 13 s rise time but a 146 s recovery time, while ${\mathrm{WO}}_{3}$ (100 ppm, 200 °C; ∼745 nm crystallite size) exhibits ∼97% response with a 12 s response time but a ∼412 s recovery time. In contrast, ${\mathrm{Al}}_{2}O_{3}$/AgO maintains response/recovery times of 11.61 s/20.43 s at 150 ppm and 250 °C without optical assistance. This performance underscores that rapid recovery (on the order of seconds), not just response magnitude, governs practical metrics such as cycle speed, short duty cycles, and drift control under repeated on/off operation [42,43].

Mechanistically, the fast kinetics of ${\mathrm{Al}}_{2}O_{3}$/AgO are consistent with AgO surface redox, including rapid Ag ↔ ${\mathrm{Ag}}_{2}$S transformations under $H_{2}$S exposure, combined with an ${\mathrm{Al}}_{2}O_{3}$ matrix that is chemically inert, thermally stable, effective at dispersing and capping AgO grains, and capable of introducing tunable grain-boundary barriers that stabilize the baseline. Controlling the pulse duration in PLD adjusts ablation energetics and, in turn, crystallite size and boundary density, which sharpens interfacial depletion/accumulation and accelerates adsorption-reaction-desorption cycles, again without resorting to photoactivation.

## 5. Potential Application Areas of the Developed Sensors

### 5.1. Industrial Safety and Leak Detection ($H_{2}S$)

The seconds-scale response and recovery demonstrated at ∼250 °C under mid-to-high-ppm $H_{2}$S exposure make these films well-suited for point-of-use monitoring in oil and gas installations, wastewater/biogas treatment plants, pulp-and-paper mills, and sewer networks, settings where short, hazardous $H_{2}$S bursts occur and rapid alarms are essential. In practice, the sensing layer can be integrated into fixed-head or portable “sniffer” units equipped with a micro-heater and a sintered or particulate inlet to protect the surface [44].

### 5.2. Process and Emissions Monitoring (${\mathrm{NO}}_{2}{\mathrm{/NO}}_{x}$ Near Source)

Near combustion equipment, boiler stacks, incinerators, and thermal processing lines, ${\mathrm{NO}}_{2}$ commonly appears in the ppm to hundreds of ppm range. A sensor combining mid-temperature operation (∼250 °C) with seconds-scale kinetics is well-positioned to track operational transients—such as fuel/air switches and ramp-up/ramp-down cycles-at the source [45].

### 5.3. Laboratory/Process Cabinets and Closed Lines

Local leak monitoring near gas manifolds or exhaust ducts—where transient concentrations can briefly rise into the mid-ppm regime-favors fast chemiresistive films operated at moderate temperatures [46].

## 6. Conclusions

This work highlights that laser pulse duration plays a decisive role in shaping both the structural and functional properties of ${\mathrm{Al}}_{2}O_{3}$/AgO thin films prepared by pulsed laser deposition (PLD). Reducing the pulse width from 10 to 6 nanoseconds causes a notable shift in the deposition dynamics: the higher peak energy of the laser enhances ablation, increases plasma density, and favors the formation of ultrafine species. Consequently, the resulting films show smaller crystallite sizes, smoother surfaces, and thinner, more uniform layers, as confirmed by XRD, AFM, and FE-SEM analyses.

Shorter pulses also strengthen the interaction between plasma and oxygen, leading to greater oxygen incorporation into the growing film, as supported by EDS results. This change in composition, combined with the effect of grain boundaries, drives both an n- to p-type conductivity transition and a bandgap widening due to quantum confinement. These modifications translate directly into improved sensing behavior: films deposited with shorter pulses demonstrate higher sensitivity toward ${\mathrm{NO}}_{2}$ (oxidizing) and $H_{2}$S (reducing) gases, owing to increased surface activity and more favorable adsorption–desorption kinetics.

Overall, controlling pulse duration provides a simple yet powerful way to tune the microstructure and electronic response of PLD-grown films, enabling the design of nanostructured sensors with enhanced stability, selectivity, and sensitivity for industrial gas monitoring. Still, a deeper understanding of selectivity requires careful evaluation of cross-sensitivity to common interfering gases such as CO, ${\mathrm{NH}}_{3}$, and particularly humidity, which will be addressed in future work. Moving from observed correlations to mechanistic insight will also call for more advanced characterization, including SKPFM, C-AFM, chemical and elemental mapping of grain boundaries, and direct probing of carrier trapping dynamics.

Looking forward, combining PLD’s strength in tailoring microstructure with the surface precision of Atomic Layer Deposition (ALD) offers a promising hybrid route to independently engineer grain morphology, heterojunctions, and catalytic surface sites [47,48]. Such an approach could push sensor performance to new levels. Upcoming research will, therefore, focus on quantifying the limit of detection (LOD) at the ppb scale, along with systematic studies on long-term stability, drift, and repeatability under realistic humidity conditions, in order to evaluate the commercial potential of these films.

## Figures and Tables

**Table 1:** XRD parameters of ${\mathrm{Al}}_{2}O_{3}$/AgO thin film.

$D_{\mathrm{ave}}$ (nm)	*D* (nm)	FWHM (Degree)	2θ (Degree)	Pulse Duration (ns)
9.703	13.300	0.702	37.953	10
	3.909	2.438	44.157	
	6.735	1.423	45.730	
	6.388	1.632	64.265	
	11.699	0.904	66.773	
	16.186	0.698	77.289	
4.093	4.018	2.330	38.778	8
	3.879	2.464	44.982	
	2.234	4.303	46.566	
	4.561	2.299	65.288	
	5.772	1.840	67.554	
2.706	1.305	6.457	38.734	6
	5.211	1.650	44.850	
	1.603	5.398	46.643	

**Table 2:** Measured values of average grain size, roughness ratio, and root mean square for ${\mathrm{Al}}_{2}O_{3}$/AgO films (AFM data).

Pulse Duration (ns)	Average Grain Size (nm)	RMS Roughness (nm)	Mean Roughness (nm)
10	27.19	3.87	2.54
8	23.95	3.13	2.04
9	17.87	2.91	1.95

**Table 3:** Weight and atomic percentages of elements in the prepared ${\mathrm{Al}}_{2}O_{3}$/AgO films.

Pulse Duration (ns)	Elements	Atomic %	Weight %
10	C	13.9	10.1
	O	51.9	29.6
	Na	6.3	7.3
	Mg	1.5	1.8
	Al	3.8	5.2
	Si	18.8	35.5
	Ca	2.2	6.4
	Ag	1.6	4.1
8	C	13.0	9.9
	O	53.7	30.6
	Na	5.6	6.6
	Mg	1.4	1.6
	Al	3.2	4.8
	Si	19.7	36.4
	Ca	2.1	6.2
	Ag	1.3	3.9
6	C	11.2	8.5
	O	55.2	31.5
	Na	5.6	6.6
	Mg	1.4	1.7
	Al	2.7	4.2
	Si	20.3	37.4
	Ca	2.1	6.3
	Ag	1.1	3.8

**Table 4:** Hall effect measurements for ${\mathrm{Al}}_{2}O_{3}$/AgO films at pulse durations of 10 ns, 8 ns, and 6 ns.

Type (p/n)	σ (Ω/cm)^−1^	$\mu_{H}$ ($m^{2}$/Vs)	$n_{H}$ (${\mathrm{1/cm}}^{3}$)	$R_{H}$ (${\mathrm{cm}}^{3}$/C)	Pulse Duration (ns)
n	$1.62\times 10^{1}$	$1.71\times 10^{3}$	$-5.93\times 10^{16}$	$-1.05\times 10^{2}$	10
p	$2.45\times 10^{0}$	$1.68\times 10^{3}$	$9.10\times 10^{15}$	$6.86\times 10^{2}$	8
p	$2.17\times 10^{0}$	$1.57\times 10^{3}$	$8.64\times 10^{15}$	$7.22\times 10^{2}$	6

**Table 5:** Sensitivity, response time, and recovery time of ${\mathrm{Al}}_{2}O_{3}$/AgO films deposited using different pulse durations (10 ns, 8 ns, and 6 ns) at various operating temperatures.

Rec. Time (s)	Res. Time (s)	${\mathrm{S-H}}_{2}$S (%)	Rec. Time (s)	Res. Time (s)	${\mathrm{S-NO}}_{2}$ (%)	Temp. (°C)	Pulse Duration (ns)
20.7	15.57	8.41	31.23	10.53	16.1	40	10
14.13	14.4	9.46	20.61	21.6	0.9	150	
17.1	19.62	37.03	17.64	14.04	37.5	250	
20.7	17.82	4.65	22.95	7.65	7.63	40	8
19.35	18.81	14.38	23.85	11.25	12.55	150	
16.02	24.84	74.20	26.28	9.27	41.7	250	
14.22	12.69	15.38	21.78	14.13	10.35	40	6
17.73	13.95	38.29	23.22	13.86	8.9	150	
20.88	31.68	50.2	20.43	11.61	55.56	250	

**Table 6:** Benchmark comparison of PLD-grown ${\mathrm{Al}}_{2}O_{3}$/AgO films versus representative thin-film ${\mathrm{platforms-CuO/SnO}}_{2}$ (composite p-n heterojunction), NiO (single-phase metal oxide), and ${\mathrm{Ag/WO}}_{3}$/rGO (hybrid multi-phase)-for ${\mathrm{NO}}_{2}$ and $H_{2}$S sensing.

Material	Gas (Concentration)	*T* (°C)	Sensitivity	Response Time (s)/Recovery Time (s)	References
${\mathrm{Al}}_{2}O_{3}$/AgO	$H_{2}$S (200 ppm)	250	74.20%	24.84/16.02	This work
${\mathrm{CuO/SnO}}_{2}$	$H_{2}$S (50 ppm)	200	85.71%	100/109	[41]
NiO	$H_{2}$S (200 ppm)	400	28.8%	108/47	[41]
${\mathrm{Ag/WO}}_{3}$/rGO	$H_{2}$S (100 ppm)	150	65.8%	8/38	[41]
${\mathrm{Al}}_{2}O_{3}$/AgO	${\mathrm{NO}}_{2}$ (150 ppm)	250	55.56%	11.61/20.43	This work
NiO	${\mathrm{NO}}_{2}$ (100 ppm)	200	45.6%	13/146	[42]
${\mathrm{WO}}_{3}$	${\mathrm{NO}}_{2}$ (100 ppm)	200	97%	12/412	[42]

## Data Availability

The original contributions presented in this study are included in the article. Further inquiries can be directed to the corresponding author.
